# Psychological and Psychiatric Consequences of Prolonged Fasting: Neurobiological, Clinical, and Therapeutic Perspectives

**DOI:** 10.3390/nu18010060

**Published:** 2025-12-24

**Authors:** Vincenzo Bonaccorsi, Vincenzo Maria Romeo

**Affiliations:** 1 Neurosinc, Via P. Bentivoglio n° 62, 95125 Catania, Italy; 2 Department of Culture and Society, University of Palermo, Viale Delle Scienze, Ed. 15, 90128 Palermo, Italy; 3 School of Psychoanalytic and Groupanalytic Psychotherapy S.P.P.G., Via Fontana n° 1, 89131 Reggio Calabria, Italy

**Keywords:** prolonged fasting, ketone bodies, β-hydroxybutyrate, neurobiological mechanisms, mood and cognitive outcomes, psychiatric risk, religious fasting, ritual fasting

## Abstract

**Background/Objectives:** Prolonged fasting—defined as voluntary abstinence from caloric intake for periods exceeding 24 h—is increasingly recognized not only as a metabolic intervention but also as a psycho-behavioral modulator. According to the 2024 international consensus, intermittent fasting encompasses diverse temporal patterns including time-restricted feeding, alternate-day fasting, and periodic fasting of multi-day duration. While metabolic benefits are well documented, the psychoneurobiological and psychiatric consequences remain incompletely characterized. This review critically appraises current evidence on the psychological and psychiatric effects of prolonged and intermittent fasting, including both secular and religious practices. **Methods:** A narrative synthesis was conducted on clinical trials, observational studies, and translational research published between January 2010 and June 2025 in PubMed, Scopus, and PsycINFO. Search terms included combinations of “prolonged fasting,” “intermittent fasting,” “psychological,” “psychiatric,” “religious fasting,” “Ramadan,” and “Orthodox Church.” Eligible studies required explicit evaluation of mood, cognition, stress physiology, or psychiatric symptoms. Data were analyzed qualitatively, with particular attention to study quality, fasting regimen characteristics, and participant vulnerability. This is a non-registered narrative synthesis drawing on clinical trials, observational studies, and preclinical evidence published between January 2010 and June 2025. **Results:** Eighty-seven studies met inclusion criteria (39 human; 48 preclinical). In metabolically healthy adults, short-term time-restricted eating and supervised prolonged fasting were associated with modest reductions in depressive symptoms and perceived stress, with small improvements in executive functioning—typically observed in small samples and with limited follow-up. Religious fasting during Ramadan and the Orthodox Christian fasting periods demonstrated similar neuropsychological effects, including greater perceived spiritual meaning and affective modulation, though cultural context played a moderating role. Potential adverse mental-health impacts included mood destabilization, anxiety exacerbation, and rare psychotic or manic decompensations in vulnerable individuals. Randomized trials reported few adverse events and no signal for severe psychiatric harm, whereas observational studies more often noted symptom exacerbations in at-risk groups. Patients with eating disorder phenotypes exhibited increased cognitive preoccupation with food and a heightened risk of behavioral relapse. Methodological heterogeneity across studies—including variation in fasting protocols, psychological assessments, and follow-up duration—limited cross-study comparability. **Conclusions:** Evidence indicates a bidirectional relationship wherein fasting may foster psychological resilience in select populations while posing significant psychiatric risks in others. Inclusion of religious fasting traditions enriches understanding of culturally mediated outcomes. To enhance rigor and safety, future studies should incorporate clinician-rated outcomes (e.g., HDRS-17, CGI-S/CGI-I), standardized adverse-event tracking using validated psychiatric terminology, and prospective safety monitoring protocols, with ≥6–12-month follow-up.

## 1. Introduction

Intermittent fasting (IF) encompasses four macro-categories recently codified by the International Consensus on Fasting Terminology—time-restricted eating (TRE), alternate-day fasting (ADF), periodic prolonged fasting (PPF ≥ 48 h) and religious/ritual fasting (RRF) [1]. Over the past decade IF—whether practiced as ≥24 h water-only fasts, 5:2 schedules, or multiday interventions—has moved from niche to mainstream, driven by its documented metabolic benefits [2,3,4]. Public and scientific interest in fasting has increased over the past decade, as reflected by the growing number of registered trials and peer-reviewed publications examining metabolic and mental-health endpoints. Yet weight-centered endpoints dominate these studies, leaving the neuropsychiatric dimension underexplored.

At the mechanistic level, metabolic switching from hepatic glycogenolysis to ketogenesis elevates circulating β-hydroxybutyrate (BHB), which crosses the blood–brain barrier and serves as an efficient neuronal substrate [5]. BHB functions as a class-I histone-deacetylase inhibitor, modulating transcription programs linked to synaptic plasticity and oxidative-stress resilience [6]. Pre-clinical work shows that intermittent or prolonged fasting boosts hippocampal brain-derived neurotrophic factor (BDNF), promotes mitochondrial biogenesis, dampens microglial activation, and recalibrates hypothalamic–pituitary–adrenal (HPA)-axis signaling—pathways tightly linked to mood and stress disorders [7,8].

Human evidence is heterogeneous. A 2023 systematic review of 15 RCTs reported that IF produced small-to-moderate reductions in depressive symptoms (Hedges g = 0.32) without major adverse events [9]; a 12-month TRE trial documented sustained improvements in Patient Health Questionnaire-9 scores alongside 8 % weight loss [10]. Conversely, a 2024 scoping review highlighted that prolonged fasts (>7 days) can heighten neuro-inflammatory markers such as IL-6 and TNF-α, raising concerns for at-risk psychiatric cohorts. Case reports still describe affective lability, anxiety spikes and, in vulnerable persons, fasting-related psychosis or mania.

Public adoption via wellness culture further blurs the boundary between therapeutic practice and disordered-eating behavior, especially among adolescents and perfectionistic young adults [11]. Thus, fasting emerges as a bidirectional tool: neuroplastic, anti-inflammatory, and potentially resilience-enhancing on one side; psychopathology-triggering when misapplied.

Objectives. This narrative review aggregates literature from 2010 to June 2025 on the psychological and psychiatric sequelae of prolonged fasting. Specifically, (i) integrating mechanistic insights connecting fasting to neural and endocrine pathways; (ii) appraising clinical data on mood, cognition, and psychiatric symptoms; (iii) delineating risk profiles and adverse events—including those linked to religious practices such as Ramadan and Orthodox fasting; and (iv) proposing evidence-based clinical guidelines and research priorities. By clarifying these domains, we aim to help clinicians harness fasting’s therapeutic potential while safeguarding vulnerable populations. This review integrates mechanistic, preclinical, and clinical perspectives to bridge biological and behavioral evidence on fasting and mental health, with emphasis on operational guidance for safe clinical translation.

## 2. Materials and Methods

### 2.1. Literature Search Strategy

A narrative search was carried out in PubMed/MEDLINE, Scopus, and PsycINFO for articles published from 1 January 2010 to 30 June 2025. The string (“prolonged fasting” OR “intermittent fasting” OR “alternate-day fasting” OR “time-restricted feeding”) AND (“psychology” OR “psychiatry” OR “mood” OR “cognition” OR “stress”) was applied to titles, abstracts, and keywords. Reference lists of retrieved papers and recent systematic reviews were screened manually to capture additional studies.

### 2.2. Eligibility Criteria (Pre-Specified)

We included studies meeting the following a priori criteria. Population (humans): Adults (≥18 years); adolescent samples were considered only when psychological/psychiatric outcomes were explicitly assessed. Interventions/exposures: Fasting paradigms, namely, time-restricted eating (TRE), alternate-day fasting (ADF), periodic/prolonged fasting, and religious/ritual fasting. Comparators: Any (active control, usual diet, parallel control, or within-subject pre–post). Human outcomes: Validated psychological/psychiatric endpoints (e.g., HDRS-17, PHQ-9, GAD-7, STAI-S, BAI; clinician-assessed relapse/recurrence) and/or stress-physiology/autonomic markers with a clear mental-health relevance (e.g., morning salivary cortisol, high-frequency heart-rate variability [HF-HRV]). Preclinical inclusion: In vivo studies directly pertinent to neurobiological pathways of mood, anxiety, or psychosis (e.g., BHB→NLRP3, BDNF/PGC-1α signaling, hypothalamic–pituitary–adrenal axis recalibration). Designs: Randomized controlled trials (RCTs), non-randomized trials, cohorts, case–control, pre–post studies, and case series (humans); in vivo experiments (preclinical). In vitro studies were excluded unless tightly anchored to converging in vivo evidence. Language/time window: English; January 2010–June 2025.

**Figure 1:**
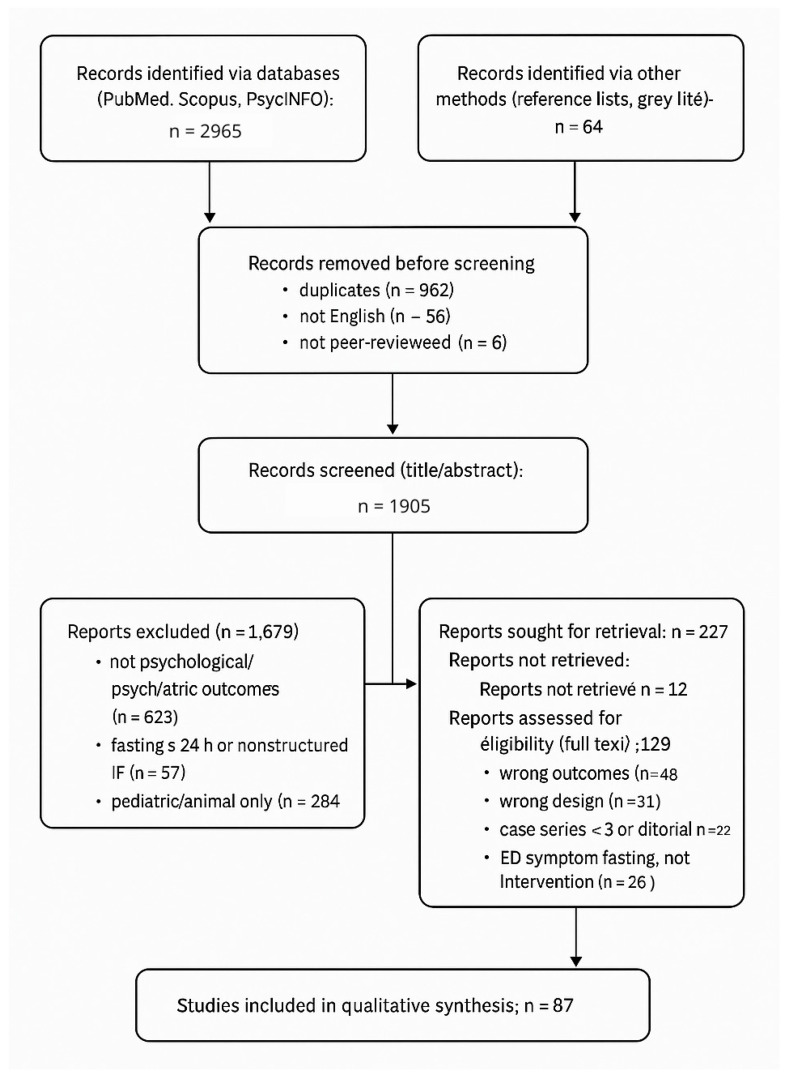
PRISMA 2020 flow diagram of the literature search and study selection for studies on prolonged or intermittent fasting and psychological or psychiatric outcomes. Records were identified in PubMed, Scopus, and PsycINFO and from additional sources (reference lists and gray literature). After removal of duplicates, non-English, and non-peer-reviewed items, titles/abstracts were screened, full texts were assessed for eligibility, and studies meeting inclusion criteria were retained. Main reasons for full-text exclusion were wrong outcomes, wrong design, case series < 3 or editorial, and reports of eating-disorder symptoms without an intervention. *n* = 87 studies were included in the qualitative synthesis. Abbreviations: IF, intermittent fasting; ED, eating disorder. See Table S1 for details.

Exclusion codes (mapped to PRISMA boxes in Figure 1) were defined as follows: wrong outcomes—absence of validated psychological/psychiatric measures or mental-health-relevant physiological markers (studies reporting only metabolic/weight outcomes without mental-health endpoints); wrong population—purely pediatric samples; severe medical cohorts without mental-health outcomes; animal/in vitro work not mechanistically relevant; wrong intervention—calorie restriction without a fasting component or diet/supplement interventions unrelated to fasting; wrong design—editorials, narrative opinions without primary data, conference abstracts without a retrievable full text; reports not retrieved—full texts unobtainable after institutional access attempts and author contact; duplicates—multiple reports from the same cohort, in which case the most complete version was retained.

### 2.3. Information Sources and Search Strategy

We searched PubMed/MEDLINE, Scopus, and PsycINFO for records published from January 2010 through June 2025. The search combined controlled vocabulary and free-text terms for fasting paradigms and mental-health outcomes (e.g., “prolonged fasting” OR “intermittent fasting” OR “time-restricted eating” OR “alternate-day fasting” OR “religious fasting”) AND (psycholog* OR psychiatr* OR mood OR anxiety OR stress OR depression). Core MeSH/keywords included fasting; intermittent fasting; time-restricted eating/feeding; alternate-day fasting; religious fasting (Ramadan, Eastern Orthodox); and mental-health terms (psychology, psychiatry, mood, depression, anxiety, stress, cognition, HPA axis, BDNF). Full Boolean strings and database-specific filters are reported in Table S1 (Supplementary Materials). We performed backward and forward citation chasing of included records. “Gray literature” was limited to reference-list screening and trial-registry checks; non-peer-reviewed materials (preprints, theses, abstracts without retrievable full texts) were excluded. Duplicates were removed using reference-manager de-duplication and manual verification.

### 2.4. Study Selection and Data Extraction

Titles/abstracts and then full texts were screened in duplicate by two reviewers working independently; disagreements were resolved by a third reviewer. For each eligible study we extracted, on pilot-tested forms, the design, population/sample, clinical status/diagnosis, fasting protocol (type, timing, duration), comparator, psychometric instruments, follow-up window, key psychological/psychiatric outcomes, and a brief design-level risk-of-bias note. These fields are summarized for human studies in Table 1 (Summary of human evidence linking prolonged fasting to key psychological outcomes) and correspond to the columns reported in Table 2. Preclinical data items included species/strain, fasting paradigm, mechanistic readouts, and behavioral endpoints when applicable. Data integrity and consistency checks were performed prior to synthesis, and reasons for exclusion at the full-text stage were recorded and are summarized in Figure 1 and detailed in Table S1.

### 2.5. Quality Assessment

Randomized controlled trials were appraised with the Cochrane RoB-2 tool, observational studies with the Newcastle–Ottawa Scale, and animal studies with the SYRCLE risk-of-bias tool. Narrative grades (low, moderate, or high risk of bias) were assigned to each study. Overall, randomized trials exhibited low-to-moderate risk of bias, mainly due to incomplete blinding and selective reporting; observational studies were moderate, with residual confounding and measurement variability; animal studies showed variable risks related to randomization and outcome assessment. These considerations informed the narrative grading of evidence strength.

### 2.6. Data Synthesis

Given heterogeneity in fasting regimens, psychiatric assessments, and endpoints—most relying on self-report instruments rather than clinician-administered interviews [23,24], a quantitative meta-analysis was not feasible. Results were therefore synthesized qualitatively, with particular attention to protocol duration, psychiatric stratification, and outcome directionality.

## 3. Results

### 3.1. Study Selection

The database and manual search identified 3029 records. After removal of duplicates (*n* = 962), non-English (*n* = 96), and non–peer-reviewed items (*n* = 6), 1905 records were screened. Of these, 227 reports were sought for full-text retrieval, 129 were assessed for eligibility, and 87 studies (39 human; 48 preclinical) were included in the qualitative synthesis (Figure 1; Table S1).

### 3.2. Characteristics of Included Human Studies

The human evidence base comprised randomized controlled trials, non-randomized trials, cohort studies, pre–post investigations, and observational studies spanning time-restricted eating (TRE), supervised prolonged fasting programs (e.g., Buchinger regimens), and religious fasting. For each study, design, sample characteristics, fasting protocol (type, timing, duration), comparator, psychometric instruments, follow-up, and outcomes are summarized in Table 2. Across human trials, intervention durations typically ranged from 4 to 12 weeks for TRE and 4–21 days for supervised prolonged fasting programs. Sample sizes were predominantly small-to-moderate (≈20–100). Primary assessment tools included PHQ-9/BDI-II (depression), GAD-7/STAI-S (anxiety), PSS (perceived stress), and selected cognitive tests (executive/working-memory tasks).

### 3.3. Synthesis of Human Outcomes

Across metabolically healthy adults, TRE and supervised prolonged fasting were associated with small improvements in depressive symptomatology and perceived stress; effect magnitudes varied by baseline symptom burden and the degree of supervision. Supervised Buchinger-type programs consistently reported reductions in State-Trait Anxiety Inventory-State (STAI-S) scores, lower morning salivary cortisol, and increases in high-frequency heart-rate variability (HF-HRV). Religious fasting yielded mixed findings: Eastern Orthodox Great Lent was associated with modest reductions in Perceived Stress Scale and Beck Anxiety Inventory scores [25], whereas during Ramadan, a higher relapse risk in bipolar disorder was observed under specific clinical conditions. Severe adverse events were uncommon but documented: isolated psychotic or manic decompensations occurred in vulnerable individuals, and restrictive-eating phenotypes tended to worsen in the absence of clinical supervision.

Overall, most human studies reported favorable changes in mood and/or perceived stress under circadian-aligned TRE or supervised prolonged fasting, whereas neutral findings were more common in longer, unsupervised, or less structured regimens.

### 3.4. Synthesis of Preclinical Mechanisms

In vivo studies converged on several pathways plausibly linking fasting to affective and stress-related outcomes: ketone-mediated signaling (e.g., β-hydroxybutyrate effects on the NLRP3 inflammasome), neurotrophic modulation (brain-derived neurotrophic factor and PGC-1α-related mitochondrial programs), hypothalamic–pituitary–adrenal axis recalibration, gut–brain interactions influencing barrier integrity and microglial activation, and immuno-oxidative attenuation. Most preclinical evidence derives from rodent models (mice/rats) employing 24–72 h fasting or TRF paradigms. While these data elucidate plausible mechanisms (e.g., BHB→NLRP3, BDNF/PGC-1α), species differences, protocol heterogeneity, and behavioral readouts limit direct clinical extrapolation.

## 4. Neurobiological and Neurochemical Effects of Prolonged Fasting

Prolonged fasting precipitates a rapid metabolic switch from hepatic glycogenolysis to ketogenesis, elevating circulating β-hydroxybutyrate (BHB), acetoacetate, and acetone within 24–48 h. During prolonged fasting or ketogenic states, circulating β-hydroxybutyrate (BHB) crosses the blood–brain barrier via monocarboxylate transporters and can supply a substantial fraction of cerebral ATP demand, in addition to its signaling functions [26]. Importantly, BHB is not merely a fuel; it operates as a pleiotropic signaling metabolite that engages host receptors and epigenetic machinery.

### 4.1. Ketone Signaling and Neurotransmission

BHB binds to hydroxycarboxylic acid receptor-2 (HCA2) on microglia, inhibiting NLRP3-inflammasome activation and downstream IL-1β release—an anti-inflammatory action germane to affective disorders [27]. As a class I histone-deacetylase inhibitor, BHB increases histone H3 acetylation at promoters of neuronal resilience genes such as BDNF and Peroxisome proliferator-activated receptor gamma coactivator 1-alpha (PGC1α) (Figure S1), thereby enhancing synaptic plasticity [28,29]. Rodent hippocampal slices exposed to physiological ketone concentrations exhibit augmented GABAergic tone and reduced glutamatergic excitability, effects reproduced in vivo by 48 h fasting [30]. These shifts underpin the anticonvulsant and anxiolytic profile of ketogenic interventions. In humans, supervised 4–21-day Buchinger fasting programs have been associated with significant reductions in perceived tension/anxiety and morning salivary cortisol, alongside increases in high-frequency heart-rate variability, indicating strengthened parasympathetic control [31].

### 4.2. Mitochondrial Biogenesis and Autophagy

Energy scarcity activates AMP-activated protein kinase (AMPK) and sirtuin-1, leading to PGC-1α-driven mitochondrial biogenesis, enhanced mitophagy, and up-regulation of antioxidant enzymes including superoxide-dismutase-2 [32]. Fasting-induced autophagic flux clears dysfunctional organelles and misfolded proteins; in neuronal cultures, a single 24 h nutrient deprivation period reduces α-synuclein aggregates via ULK1 activation [33].

### 4.3. Neuroendocrine and Autonomic Recalibration

Acute fasts elevate cortisol and catecholamines, yet repeated cycles dampen basal ACTH and enhance vagal tone, suggesting a recalibrated hypothalamic–pituitary–adrenal axis (Figure 1) conducive to stress resilience [34]. Concomitantly, adiponectin—a metabolic hormone with AdipoR1-AMPK-mediated antidepressant effects—increases both peripherally and centrally, correlating with improvements in mood scores [35].

### 4.4. Gut–Brain Axis Modulation

Time-restricted feeding paradigms remodel gut microbial communities, enriching butyrate-producing taxa such as Roseburia and Faecalibacterium. These metabolites fortify blood–brain barrier integrity and dampen microglial activation, thereby influencing anxiety-like behavior in mice [36,37,38]. A pilot crossover study reported parallel shifts in microbial α-diversity and reductions in perceived stress after two weeks of 16:8 fasting in healthy adults [39].

### 4.5. Immuno-Oxidative Landscape

Meta-analytical evidence indicates that fasting interventions ≥4 weeks reduce high-sensitivity C-reactive protein, Tumor Necrosis Factor alpha (TNF-α), and Interleukin-6 (IL-6) by 10–30%, with concomitant down-regulation of NF-κB-responsive transcripts [40]. These changes mirror clinical improvements in depressive symptomatology and may constitute a mechanistic bridge linking metabolic and mood benefits.

Taken together, ketone-mediated signaling, mitochondrial rejuvenation, endocrine recalibration, microbiota reshaping, and immuno-oxidative dampening converge to form a neurobiological substrate through which prolonged fasting can influence cognition, emotion, and psychiatric vulnerability. Inter-individual variability in these cascades—driven by genetics, sex, circadian alignment, and psychiatric history—highlights the need for personalized protocols and mechanistic endpoints in future trials.

## 5. Psychological Outcomes

### 5.1. Mood

Emerging evidence positions prolonged or intermittent fasting (IF) as a double-edged sword in the affective domain. On one side, metabolic switching and the attendant rise in β-hydroxybutyrate (BHB) trigger neurotrophic, anti-inflammatory, and monoaminergic cascades that may convey antidepressant and anxiolytic benefits; on the other, caloric deprivation can destabilize mood in susceptible individuals, particularly when fasting is unsupervised or prolonged. Below, we synthesize data across preclinical models, mechanistic biomarker studies, and clinical trials, highlighting moderators that tilt the fasting–mood relationship toward resilience or risk.

Rodent models demonstrate that 24–48 h water-only fasts increase hippocampal BDNF and activate the CREB–PGC-1α axis, yielding behavioral antidepressant-like phenotypes in forced-swim and tail-suspension tests [41]. Translationally, association between circulating BHB and proBDNF mRNA in human peripheral mononuclear cells, correlating with mood elevation [42]. A 2022 randomized controlled trial (RCT) in 93 adults with mild-to-moderate major depressive disorder (MDD) reported that a 4-week 16:8 IF protocol reduced Hamilton Depression Rating Scale (HDRS-17) scores by −4.8 ± 1.3 points versus −1.9 ± 1.1 in an isocaloric three-meal control, accompanied by increased serum BDNF and lower high-sensitivity C-reactive protein [43]. These findings align with a recent network meta-analysis [44] showing small-to-moderate effect sizes (Hedges g = 0.29) for depressive symptom reduction across six IF randomized controlled trials (Figure 1). Representative randomized trials reported small-to-moderate symptom reductions on clinician-rated (e.g., HDRS-17) and self-report measures (e.g., STAI-S, PHQ-9), sometimes accompanied by BDNF shifts. Conversely, null effects on BDI-II/POMS at 12 months under less intensive TRE underscore heterogeneity by protocol duration, baseline symptoms, and assessment modality.

### 5.2. Anxiety

Fasting modulates hypothalamic GABA/glutamate ratios and attenuates NLRP3-inflammasome activity, mechanisms implicated in anxiolysis (Figure 1). In mice, a 24 h fast reduces anxiety-like behavior and improves memory via brain-wide reductions in caspase-1 activity (a key regulator of IL-1β maturation) [45]. Clinically, healthy adults undergoing a 10-day Buchinger water fast exhibited significant reductions in State-Trait Anxiety Inventory-State (STAI-S) scores [46]. However, anxiety outcomes across trials are heterogeneous, partly due to differing baseline anxiety levels and assessment tools.

Psychological responses to fasting vary markedly by sex, psychiatric history, and hormonal milieu. Women with pre-existing MDD or bipolar II disorder appear particularly vulnerable to mood destabilization during calorie restriction [47]. Fasting-induced hypoglycaemia, cortisol surges, and sleep fragmentation may jointly precipitate irritability, anergia, or mixed affective states. In an observational cohort of 1 214 Ramadan fasters, 7.4% of participants with a prior mood-disorder diagnosis experienced clinically significant symptom exacerbation [48].

Sleep disruption is a salient mediator: in a randomized pilot trial, early time-restricted eating shifted sleep timing in late sleepers; independently, selective REM-sleep suppression increases next-day negative affect [49]. Moreover, fasting can cue maladaptive cognitive–emotional schemas in individuals with dieting history, potentiating rumination and guilt, which are transdiagnostic risk factors for depression [50].

Collectively, data suggest a U-shaped relationship wherein moderate, circadian-aligned fasting confers modest affective benefits in metabolically healthy adults, while aggressive or prolonged restriction heightens risk in vulnerable groups. Screening for mood-disorder history, sleep quality, and stress load is therefore essential before prescribing fasting interventions. We recommend (i) starting with ≤14 h fasting windows, (ii) maintaining consistent bedtime to preserve REM sleep, (iii) monitoring glucose and cortisol in patients with affective disorders, and (iv) integrating cognitive–behavioral strategies to mitigate guilt-based cognitions. Large-scale, stratified RCTs with clinician-rated endpoints and ≥6-month follow-up are urgently needed to define safety boundaries.

### 5.3. Stress Correlations

Fasting elicits a biphasic stress response. Acutely (<24 h), glycogen depletion activates the hypothalamic–pituitary–adrenal (HPA) axis, elevating ACTH and cortisol to mobilize glucose and free fatty acids (Figure 1), whereas with repeated cycles, homeostatic recalibration emerges, characterized by blunted basal cortisol, enhanced vagal tone, and lower heart-rate-variability indices of sympathetic drive [51]. In middle-aged mice, eight weeks of alternate-day fasting normalized diurnal corticosterone, up-regulated hippocampal glucocorticoid-receptor phosphorylation, and improved stress-coping in forced-swim and tail-suspension tests [52]. Translationally, in adults completing a 10-day Buchinger water fast, morning salivary cortisol decreased (≈18%) and high-frequency HRV increased, consistent with strengthened parasympathetic control and stress-system recalibration [53].

### 5.4. Religious Fasting

Structured religious fasts (e.g., those aligning feeding to circadian windows or imposing abstention from specific macronutrients) plausibly recruit similar stress-system adaptations, while differing in intent, oversight, and social context. At the immune–neuroendocrine interface, fasting paradigms comparable to religious regimens have been associated with 10–30% reductions in circulating IL-6 and TNF-α and with down-regulation of NF-κB–responsive transcripts, changes that track improvements in perceived stress and mood [54]. Mechanistically, ketosis emerging in longer fasts can exert ketone-mediated anti-inflammatory effects—β-hydroxybutyrate directly inhibits the NLRP3 inflammasome—thereby attenuating innate-immune activation relevant to stress responsivity and affective outcomes [55]. Human fasting programs with supervised protocols further illustrate feasibility and safety signals (cortisol reduction, HRV shifts), offering a translational bridge to religious contexts where adherence, sleep timing, and communal practices may modulate stress-related benefits [56]. Analyses of Ramadan studies should account for confounders such as altered sleep timing, caffeine intake, medication dosing schedules (e.g., lithium), and daily routine changes, which can independently influence mood or relapse risk.

### 5.5. Severe Adverse Events

#### 5.5.1. Eating Disorders

Structured fasting overlaps phenomenologically with restrictive-eating pathology. Individuals with anorexia nervosa, atypical anorexia, or high-functioning orthorexia often employ extended fasts as compulsive rituals, reinforcing dopaminergic reward dysregulation, interoceptive mistrust, and perfectionism-driven hyper-control [57]. Epidemiological surveys indicate that up to 38% of female college students practicing intermittent fasting meet threshold or sub-threshold criteria for an eating disorder [58]. Wellness-culture promotion of IF may thus mask prodromal ED behaviors. Pre-intervention screening for dietary rigidity, body-image concern, and obsessive–compulsive traits is imperative; where such red flags surface, fasting should be deferred or implemented only within a specialized ED framework.

#### 5.5.2. Psychosis

Although uncommon, psychotic decompensations during prolonged fasting have been reported—for example, recurrent paranoid-hallucinatory episodes immediately following week-long fasts in a patient with anorexia nervosa—and Ramadan fasting has been associated with higher relapse risk in bipolar disorder in observational data. Mitigation strategies include maintaining antipsychotic dosing schedules, encouraging nocturnal caloric intake to stabilize glycaemia, and providing close psychiatric follow-up during the fasting period (see Table S1 in the Supplementary Materials for discontinuation criteria).

In support of the synthesis above, Table 2 provides a detailed, study-level overview of human investigations (2010–2025) across fasting paradigms (Buchinger fasting, TRE/eTRE, FMD, Ramadan), including sample characteristics, protocols, psychometric measures, follow-up, outcomes, and design-level risk of bias.

## 6. Clinical Integration and Ethical Considerations

### 6.1. Therapeutic Fasting in Psychiatric Populations

Early-phase studies have begun testing fasting-mimicking diets (FMDs), alternate-day fasting, and 16:8 time-restricted eating (TRE) as adjuncts to standard care in major depressive disorder, post-traumatic stress disorder, and mild cognitive impairment. A randomized controlled pilot trial in adults with major depression (*n* = 20) showed that adding three monthly cycles of a fasting-mimicking diet to structured psychotherapy improved self-esteem and psychological quality of life versus psychotherapy alone, with comparable depressive-symptom reduction across groups [59]. An ongoing randomized controlled trial is comparing time-restricted eating with a Mediterranean-diet control for symptom reduction and quality of life in bipolar disorder, with rater-blinded assessments across 8–15 months. Despite these encouraging signals, fasting remains contraindicated or high-risk in patients with active psychosis, severe impulse-control disorders, borderline personality disorder with suicidality, and uncontrolled substance use.

Clinical checklist (monitoring and safety):

Initial assessment (week 0): Psychiatric history; current diagnoses; ED screening; suicidality; medications; metabolic panel (electrolytes, glucose/ketones if applicable); BMI and weight trajectory; sleep and caffeine use.
Protocol start: For at-risk patients, prefer ≤14 h fasting windows; maintain consistent sleep timing; avoid abrupt medication schedule changes.
Follow-up (every 2 weeks): Clinician-rated scales (e.g., HDRS-17, CGI-S/CGI-I), self-report (PHQ-9/GAD-7/PSS), adverse-event checklist (psychiatric and somatic), weight/BMI, basic labs if indicated.
Discontinuation criteria (“STOP”): ≥5% weight loss in <4 weeks, emergent suicidality, psychotic or manic symptoms, severe anxiety/agitation, electrolyte derangements, or clinically significant sleep disruption non-responsive to adjustments.
Coordination: Multidisciplinary oversight (psychiatry, nutrition/dietetics, internal medicine/endocrinology), with shared decision-making and informed consent emphasizing uncertain long-term efficacy.

### 6.2. Ethical and Clinical Governance

Implementing fasting interventions in psychiatric settings raises distinct ethical imperatives. Informed consent must articulate (i) uncertain long-term efficacy, (ii) potential for mood destabilization or metabolic derangement, and (iii) the right to withdraw without prejudice. High-risk conditions include active eating disorders (anorexia nervosa, bulimia nervosa, severe ARFID), unstable mood or psychotic disorders, and patients under active psychopharmacological treatment with narrow therapeutic windows or dosing-time sensitivity (e.g., lithium, sedative-hypnotics); in such cases, fasting should be avoided or undertaken only with close medical supervision. Contraindication screening should encompass active eating disorders, BMI < 18.5 kg m^−2^, pregnancy, insulin-treated diabetes, cognitive impairment affecting decision-making capacity, and unstable cardiovascular or renal disease. A multidisciplinary team—psychiatrist, dietitian, internist/endocrinologist, and where available, clinical psychologist—should co-design the protocol and oversee monitoring.

Risk–benefit assessment ought to be continuous: laboratory review at baseline and week 4, structured mood and suicidality scales every fortnight, and caregiver check-ins for patients with limited insight. Ethics-committee oversight is advised for research applications, with rapid-response plans for adverse psychiatric events. Finally, fasting must remain a reversible, individualized adjunct rather than a one-size-fits-all replacement for evidence-based pharmacotherapy or psychotherapy.

## 7. Research Gaps and Future Directions

Despite burgeoning interest, the evidence base for prolonged fasting in psychiatric contexts remains nascent. Priority areas include the following:

(1) Protocol standardization. Heterogeneous fasting windows (12–72 h), feeding macronutrient composition, and cycle duration hinder comparability; consensus statements mirroring exercise prescription guidelines are needed.
(2) Psychiatric stratification. Forthcoming trials should stratify by diagnostic category, illness phase, and psychotropic medication to delineate differential risk–benefit profiles.
(3) Long-term safety and efficacy. Most studies last ≤12 weeks; prospective cohorts ≥12 months are crucial to detect delayed metabolic, endocrine, or affective sequelae.
(4) Mechanistic biomarkers. Integrative omics (metabolomics, epigenomics), inflammatory panels, and digital phenotyping of mood/sleep could clarify mediators and moderators of response.
(5) Gut–brain axis. Controlled feeding studies isolating fiber and prebiotic intake will determine how microbiota shifts contribute to affective and cognitive outcomes.
(6) Sex and age effects. Given hormonal modulation of HPA and reward circuitry, sex-specific and developmental-stage analyses are imperative.
(7) Digital-assisted monitoring. Wearable glucose/ketone sensors and app-based mood tracking may enhance safety and adherence, yet require validation in psychiatric samples.
(8) Expectancy bias mitigation. Employ blinded outcome assessors, neutral framing, and objective biomarkers (e.g., inflammatory panels, HRV, digital sleep metrics) to reduce expectancy and measurement biases.
(9) Standardized adverse-event reporting. Adopt validated psychiatric terminology and structured AE taxonomies with severity/relatedness grading, enabling cross-study comparability and safety-signal detection.

Addressing these gaps will enable evidence-based, personalized fasting protocols and inform clinical guidelines.

## 8. Conclusions

Prolonged fasting represents a potent metabolic intervention whose neurobiological ripple effects extend to mood, cognition, stress physiology, and—under specific conditions—psychiatric risk. The balance between benefit and harm pivots on protocol design, individual vulnerability, and clinical oversight. Current data support cautious, short-duration, nutrient-replete fasting as an adjunct in select psychiatric populations, provided rigorous screening and monitoring frameworks are in place. Future research should adopt standardized protocols, stratify by diagnosis, sex, and developmental stage, and incorporate mechanistic biomarkers to establish causal pathways.

Large, multisite randomized controlled trials with ≥12-month follow-up—integrating digital phenotyping, wearable biosensors, and health-economic analyses—are now needed to verify efficacy, durability, and scalability. In parallel, real-world implementation studies should evaluate clinician training, patient acceptability, and equity of access across diverse healthcare settings. Future trials should prioritize clinician-rated endpoints, standardized psychiatric adverse-event reporting, and longer follow-up (≥6–12 months) to substantiate durability of mental-health benefits and refine safety boundaries across diagnostic strata.

## Figures and Tables

**Table 1:** Summary of human evidence linking prolonged fasting to key psychological outcomes.

Domain	No. of Human Studies	Predominant Designs (n)	Net Direction of Effect	Overall Quality	Representative Studies (Author, Year)	Sample Size/Population	Fasting Protocol	Follow-Up Duration	Key Findings
Mood (Depression/Anxiety)	18 (12 RCT, 6 Obs)	RCT (12), Prospective (3), Cross-sectional (3)	Mixed positive	Moderate	[11,12]	*n* = 93 adults with MDD; college students	16:8 IF for 4 weeks; ad lib fasting	4–8 weeks	↓ Depression scores; ↑ BDNF; ↑ guilt in perfectionists
Cognitive Function	11 (6 RCT, 5 Obs)	RCT (6), Crossover (2), Cohort (3)	Positive acute; neutral/negative chronic	Moderate	[13]	*n* = 60 older adults; healthy young/overweight adults	16:8 IF for 3 months; 48 h water-only fast/TRF	12 weeks (TRE)/48 h (acute)	↑ Working memory acutely; mixed effects long-term; sleep changes may blunt mood
Stress Physiology/HPA Axis	9 (4 RCT, 5 Obs)	RCT (4), Cohort (5)	↓ Basal cortisol, ↑ HRV	Low–Moderate	[14,15]	*n* = 1422 adults in supervised fasting programs	10–21 day supervised periodic fasting; TRE variants	10–21 days	↓ Cortisol; ↑ HRV; improved stress-system resilience
Eating-Disorder Risk	7 (0 RCT, 7 Obs)	Cross-sectional (5), Cohort (2)	↑ Restrictive behaviors	Low	[16]	*n* = 738–8000 college/clinical samples	Varied unsupervised IF and extended fasts	Cross-sectional or 4-week longitudinal	↑ Restrictive behaviors; relapse risk in vulnerable phenotypes
Psychosis/Manic Switch	4 (0 RCT, 4 Case/Cohort)	Case reports (3), Cohort (1)	Rare but severe exacerbations	Very Low	[17,18]	3 case reports; one community cohort (*n* ≈ 1214)	Ramadan/Lent-aligned fasts; prolonged fasts	2–12 weeks	Rare mania/psychosis during religious or prolonged fasts; supervision advised

Legend: Detailed summary of representative human studies assessing the psychological and psychiatric outcomes of prolonged or intermittent fasting. For each functional domain, predominant study designs, population characteristics, fasting protocols, and observed effects are reported. Study quality reflects narrative GRADE-based assessments. Abbreviations: IF = intermittent fasting; RCT = randomized controlled trial; TRF = time-restricted feeding; HPA = hypothalamic–pituitary–adrenal; HRV = heart-rate variability; MDD = major depressive disorder; BDNF = brain-derived neurotrophic factor. Symbols: ↑, increase; ↓, decrease.

**Table 2:** Human studies (2010–2025) assessing psychological and psychiatric outcomes of fasting interventions.

Study (First Author, Year)	Country	Design	Population/Sample	Clinical Status/Diagnosis	Fasting Protocol (Type and Duration)	Comparator/Control	Psychological/Psychiatric Instruments	Evaluation Method (Clinician-Rated vs. Self-Report)	Follow-Up Duration	Adverse-Event Documentation (Psychiatric/Somatic)	Key Outcomes	Risk of Bias	DOI
[14]	Germany	Observational pre–post cohort (clinic registry)	Adults attending Buchinger Wilhelmi clinics; *n* = 1422; mixed indications; mean age ~ 56 y; both sexes	General medical outpatients; no primary psychiatric diagnosis required	Buchinger periodic fasting, 4–21 days; ~250–500 kcal/day broths/juices + ≥2.5 L water; light activity	None (within-subject pre–post)	Self-rated emotional/physical well-being; subjective health-complaints log	Self-report	During fast and end-of-fast (up to 21 days)	<1% adverse events recorded; psychiatric AEs not specified	Marked improvements in self-reported well-being	Observational (no control)	https://doi.org/10.1371/journal.pone.0209353
[19]	USA	Randomized clinical trial (parallel-arm)	Adults with obesity; *n* = 90; mean BMI 39.6; 80% female	Obesity; no severe chronic disease	Early time-restricted eating (07:00–15:00) + energy restriction; 14 weeks	Control eating window (≥12 h) + energy restriction	POMS subscales (fatigue–inertia, vigor–activity, depression–dejection)	Self-report	14 weeks	Not specified (psychiatric AEs)	Greater improvements in mood indices vs. control; diastolic BP improved	RCT (moderate sample)	https://doi.org/10.3390/nu11061234.
[20]	USA	Randomized controlled trial (12-month, three-arm; secondary analysis)	Adults with obesity; *n* = 90; 12-month intervention	Obesity	8 h TRE (12:00–20:00) without calorie counting; 12 months	Daily calorie restriction (25% ER) and no-intervention control	BDI-II; POMS; SF-36 (Quality of Life)	Self-report	12 months	Not specified (psychiatric AEs)	No significant changes on BDI-II/POMS/SF-36 vs. controls; trend toward increased vitality	RCT; secondary analysis; adequate follow-up	https://doi.org/10.7326/M23-0052.
[13]	Lithuania	Controlled clinical trial	Overweight women; ~46	Overweight/obesity; otherwise healthy	48 h total water-only fast	Non-fasting/standard intake	Subjective stress ratings; mood scales; EEG; cognitive and psychomotor tests	Self-report (stress/mood); objective EEG	48 h (acute)	Not specified	Moderate stress increase; no significant mood/cognition effects	Short-term clinical trial; small sample	https://doi.org/10.1016/j.bbr.2017.10.028
[21]	Italy	Randomized controlled pilot trial	Adults with MDD; *n* = 20 (pilot)	Major depressive disorder (outpatients)	Fasting-mimicking diet (FMD), three monthly 5-day cycles; adjunct to psychotherapy	Structured psychotherapy alone	Clinician- and self-rated depression severity; self-esteem; psychological QoL	Both (clinician-rated + self-report)	~3 months	Not specified	Improved self-esteem and psychological QoL vs. psychotherapy; depressive-symptom reduction comparable	Pilot RCT; small sample	https://doi.org/10.1002/jclp.22971
[3]	USA	Cross-sectional (Healthy Minds Study)	US college students; n > 8000 (2016–2020)	General college population	Self-reported fasting behavior (observational)	N/A (associational)	PHQ-9; GAD-7; SCOFF	Self-report	None (cross-sectional)	N/A (observational, no intervention)	Fasting behavior associated with higher odds of depression, anxiety, ED symptoms, SI, NSSI	Observational; residual confounding likely	https://doi.org/10.1056/NEJMra1905136.
[22]	Morocco	Observational clinical report (letter)	Psychiatric outpatients with bipolar disorder	Bipolar disorder	Ramadan fasting (~29–30 days; dawn-to-sunset)	Non-fasting bipolar outpatients; adjusted for sleep, caffeine, lithium levels	Clinician-assessed relapse/recurrence	Clinician-rated	1 month (Ramadan)	Relapse/recurrence events tracked (psychiatric)	Higher recurrence among fasters vs. non-fasters (adjusted)	Observational; brief report	https://doi.org/10.1002/wps.20113

Legend. For each study, design, population/sample, clinical status/diagnosis, fasting protocol, comparator/control, psychometric instruments, evaluation method (clinician-rated vs. self-report), follow-up duration, adverse-event documentation (psychiatric/somatic), key outcomes, design-level risk of bias, and DOI are reported. Abbreviations: TRE, time-restricted eating; ER, energy restriction; FMD, fasting-mimicking diet; ED, eating disorder; PHQ-9, Patient Health Questionnaire-9; GAD-7, Generalized Anxiety Disorder-7; POMS, Profile of Mood States; QoL, quality of life; NSSI, non-suicidal self-injury.

## Data Availability

No new data were created or analyzed in this study. Data sharing is therefore not applicable.

## Supplementary Materials

The following supporting information can be downloaded at: https://www.mdpi.com/article/10.3390/nu18010060/s1, Table S1: PRISMA-style Search Strategy and Study Selection Flow (Corrected); Figure S1: This table reports the database-specific search strategies (2010–June 2025) and the numerical PRISMA flow corresponding to Figure 1 for studies on prolonged/intermittent fasting and psychological or psychiatric outcomes.

## Abbreviations

The following abbreviations are used in this manuscript:

BDNF	Brain-Derived Neurotrophic Factor
IF	Intermittent Fasting
TRE	Time-Restricted Eating
ADF	Alternate-Day Fasting
RRF	Religious/Ritual Fasting
BHB	β-Hydroxybutyrate
HPA	Hypothalamic–Pituitary–Adrenal (axis)
HCA2	Hydroxycarboxylic Acid Receptor 2 (HCAR2; GPR109A)
AMPK	AMP-Activated Protein Kinase
TNF-α	Tumor Necrosis Factor-Alpha
IL-6	Interleukin-6
RCT	Randomized Controlled Trial
MDD	Major Depressive Disorder
HDRS-17	17-Item Hamilton Depression Rating Scale
STAI-S	State-Trait Anxiety Inventory-State subscale
PPF	Periodic Prolonged Fasting
IL6	Interleukin-6
TNFα	Tumor Necrosis Factor-Alpha
ATP	Adenosine Triphosphate
IL1β	Interleukin-1 Beta
H3	Histone H3
PGC1α	Peroxisome Proliferator-Activated Receptor Gamma Coactivator 1-Alpha
GABA	Gamma-Aminobutyric Acid
ULK1	Unc-51-Like Autophagy-Activating Kinase 1
ACTH	Adrenocorticotropic Hormone
NF-κB	Nuclear Factor Kappa-Light-Chain-Enhancer of Activated B Cells
PRISMA	Preferred Reporting Items for Systematic Reviews and Meta-Analyses
ED	Eating Disorder
CREB-PGC	cAMP Response Element-Binding Protein–PGC-1 Axis
NLRP3	NOD-, LRR- and Pyrin Domain-Containing Protein 3 (Inflammasome)
IL-1β	Interleukin-1 Beta
REM	Rapid Eye Movement
NMDA	N-Methyl-D-Aspartate
TRF	Time-Restricted Feeding
HRV	Heart-Rate Variability
FMD	Fasting-Mimicking Diet
PTSD	Post-Traumatic Stress Disorder
CAPS-5	Clinician-Administered PTSD Scale for DSM-5
BMI	Body Mass Index
CNS	Central Nervous System.
PGC-1α	Peroxisome Proliferator-Activated Receptor Gamma Coactivator 1-Alpha
