# Supplementary material for: Psychological and Psychiatric Consequences of Prolonged Fasting: Neurobiological, Clinical, and Therapeutic Perspectives
Source: Nutrients. 2025 Dec 24;18(1):60. doi: 10.3390/nu18010060 (PMC12787741; doi:10.3390/nu18010060)
Supplement: Supplementary file 1 [file nutrients-18-00060-s001.zip › nutrients-3790833REV2 Table_S1_PRISMA.pdf]

Table S1. PRISMA-style Search Strategy and Study Selection Flow (Corrected)

This table summarizes the corrected PRISMA flow diagram values reported in the manuscript entitled 'Psychological and Psychiatric Consequences of Prolonged Fasting: Neurobiological, Clinical, and Therapeutic Perspectives'. The flow aligns with Figure 1 and section §3.1 of the main text.

Stage / Decision	n
Records identified via databases (PubMed, Scopus, PsycINFO)	2,965
Records identified via other methods (reference lists, grey literature)	64
Records removed before screening – duplicates	962
Records removed before screening – not English	96
Records removed before screening – not peer-reviewed	6
Records screened (title/abstract)	1,905
Reports excluded at screening	1,678
Reports not retrieved (at screening stage)	11
Reports sought for retrieval (eligibility)	227
Reports not retrieved (eligibility)	12
Reports assessed for eligibility (full text)	129
Full-text exclusions – wrong outcomes	48
Full-text exclusions – wrong design	31
Full-text exclusions – case series <3 or editorial	22
Full-text exclusions – ED symptom fasting, not intervention	28
Studies included in qualitative synthesis	87
