# Supplementary figures and images for: Psychological and Psychiatric Consequences of Prolonged Fasting: Neurobiological, Clinical, and Therapeutic Perspectives
Source: Nutrients. 2025 Dec 24;18(1):60. doi: 10.3390/nu18010060 (PMC12787741; doi:10.3390/nu18010060)

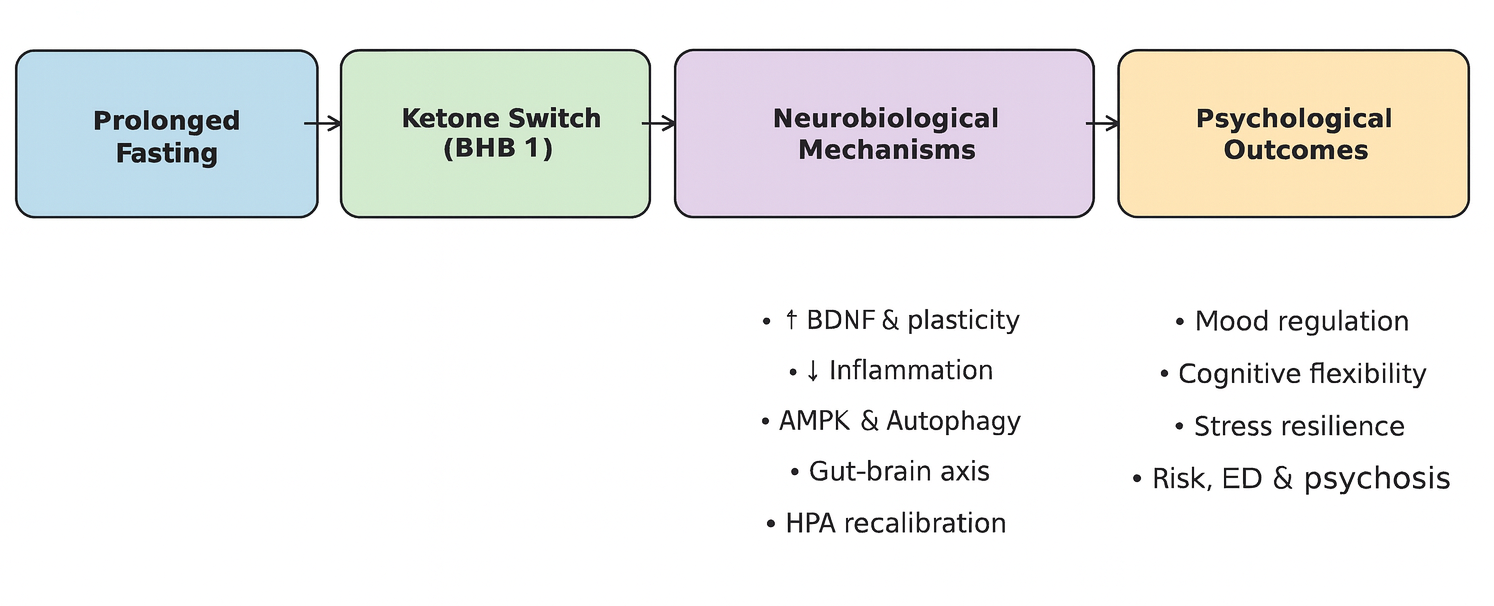

Supplement: Supplementary file 1 [file nutrients-18-00060-s001.zip › nutrients-3790833REV2 figure S1 .png]
